# Facilitators of and Barriers to Implementation of a Tablet-Based Digital Health Program for Colorectal Cancer Screening in Primary Care: Qualitative Pragmatic Implementation Study

**DOI:** 10.2196/74206

**Published:** 2026-03-13

**Authors:** Mark Cromo, Aliza Randazzo, David P Miller Jr, Kristie L Foley, Ajay Dharod, Anna C Snavely, Elena Wright, Holly Brower, Mark Dignan

**Affiliations:** 1 College of Medicine Department of Internal Medicine University of Kentucky Lexington, KY United States; 2 Department of Implementation Science Wake Forest University School of Medicine Winston-Salem, NC United States; 3 Department of Internal Medicine Department of Implementation Science Wake Forest University School of Medicine Winston-Salem, NC United States; 4 Department of Biostatistics and Data Science Wake Forest University School of Medicine Winston-Salem, NC United States; 5 School of Business Wake Forest University Winston-Salem, NC United States

**Keywords:** clinical decision support, mobile health technology, electronic health records, medical informatics applications, primary health care

## Abstract

**Background:**

Colorectal cancer (CRC) incidence and mortality rates continue to be elevated even though effective screening methods are widely available. To increase CRC screening in primary care practices, our team developed a tablet-based digital health program (mPATH) designed to identify patients needing CRC screening, provide education, and empower patients to request a screening test via the program.

**Objective:**

This study aimed to qualitatively assess facilitators of and barriers to implementing and maintaining mPATH in primary care clinics.

**Methods:**

In a pragmatic implementation trial, clinics were randomized to receive only in-person training and technological support via phone or email (low touch) or added levels of support, such as at-elbow support during launch, regular check-ins, memos, and reports (high touch). After implementation and data collection were concluded, we conducted telephone interviews with health care providers, clinic managers, and front desk and nursing staff recruited from 8 primary care clinics of varying sizes and with varying degrees of implementation of mPATH. The interviews were designed to collect perceived facilitators of and barriers to using mPATH. All interviews were administered via telephone by a single project staff member with no prior contact with participants. Interviews were audio-recorded, and 2 study team members independently coded each interview transcript and developed a codebook to identify meaningful categories in the dataset. The coders met periodically to resolve discrepancies. Data within each category were abstracted and synthesized into themes. Themes were determined inductively by prevalence and salience in the data per the principles of thematic analysis.

**Results:**

A total of 33 interviews were completed between September 2021 and April 2023 with health care providers (n=8, 24.2%), clinic managers (n=9, 27.3%), nursing staff (n=8, 24.2%), and front desk staff (n=8, 24.2%). Interviews averaged 26.7 (SD 4.9) minutes. Barriers and facilitators identified varied among clinic sites and by clinic role. Overall, the primary factors supporting the implementation of mPATH were health care provider and staff buy-in, perceived potential time savings, and workflow improvement. The primary barriers identified were perceived lack of need for the program and technical issues. There was no significant indication that clinic size or randomization to low- or high-touch training and support played a role in the decision to continue or stop using the program.

**Conclusions:**

Implementation of a tablet-based CRC screening tool in primary care practices is feasible with health care provider and staff buy-in and validation of potential time savings and workflow improvements but may be limited by perceived lack of need for the program and technical issues.

## Introduction

Colorectal cancer (CRC) is the third most common cancer diagnosed among men (15.1/100,000) and women (10.7/100,000) in the United States, and it is the second leading cause of cancer-related death [1,2]. In 2025, an estimated 154,000 Americans will be diagnosed with CRC, and more than 52,000 will die from CRC [1]. The US Preventive Services Task Force recommends that average-risk adults be screened for CRC starting at the age of 45 years using any of several efficacious tests [3]. Unfortunately, although CRC screening rates have increased in recent decades, they remain relatively low [4] at approximately 70% and fall below the goal of 80% in every community promoted by the American Cancer Society National Colorectal Cancer Roundtable [5].

Multilevel barriers contribute to low CRC screening rates. Some patients are unaware of screening methods, and those aware of stool-based screening may be reluctant to provide stool samples [6,7]. For colonoscopy, patients report difficulty completing the preparation and may have other barriers such as cost, the need to take time off work, and arranging for transportation [8]. Decision aids have been developed and tested for prostate cancer, lung cancer, and CRC screening to assist health care providers and patients in identifying suitable screening methods [9-12], but primary care clinicians report that they lack the time needed for shared decision-making [13-16].

With the goal of creating a decision aid that addresses the patient, health care provider, and system barriers to CRC screening and requires minimal time from health care providers to implement, our group developed and tested a tablet-based app (mPATH) that identifies patients overdue for CRC screening, provides education, and allows them to request a screening test via the program. In a prior randomized controlled trial conducted in community-based primary care practices, mPATH doubled the proportion of patients who completed CRC screening [17].

To determine the optimal strategy for implementing the app in routine care, we then conducted a pragmatic cluster randomized type III hybrid implementation-effectiveness trial in 18 community-based primary care practices to evaluate a “low-touch” vs “high-touch” bundle of implementation strategies [18]. Low-touch clinics received an on-site training session on use of the app and access to ongoing technical support. High-touch clinics received the same support plus at-elbow support on the day the program was launched in the clinic, as well as periodic coaching calls throughout the 6-month implementation period and regular audit and feedback reports on program use. We observed initial moderate use of the program, which decreased over time with no difference by implementation strategy [19-21]. We also observed variation in use of the program across practices and staff. To identify and better understand the factors that led to higher or lower use of the program, we conducted in-depth interviews with clinicians and staff who participated in the trial.

## Methods

### Overview of the mPATH Program

On the basis of formative research with clinical staff and health care providers [18], the mPATH app was divided into 2 separate tablet programs: mPATH-CheckIn and mPATH-CRC. mPATH-CheckIn was provided to patients by front desk staff at check-in to be completed in the waiting room. mPATH-CheckIn included the health system’s mandated screening items for depression, fall risk, and intimate partner violence. An application programming interface connection to the electronic health record (EHR) imported CRC screening history. For age-eligible patients with no record of current CRC screening, mPATH-CheckIn asked additional questions to determine whether screening had been completed outside the health system and whether the patient had risk factors that would change screening recommendations. Those patients indicated as eligible or due for screening on mPATH-CheckIn were then provided with a separate tablet (mPATH-CRC) by nursing staff when they transitioned from the waiting room to an examination room, which displayed a screening decision aid video and closed by allowing patients to request a screening test, either a fecal immunochemical test or a colonoscopy. mPATH-CRC then immediately sent an EHR in-basket message to the health care provider, alerting them that the patient had used the program and summarizing the outcome. When the health care provider arrived in the examination room, a pop-up alert let the provider order the requested CRC screening test with minimal clicks.

### Recruitment

We intentionally selected 8 primary care clinics from the 18 enrolled in the randomized trial described previously [20], varying by practice size, participant group, and degree of mPATH adoption, with the aim to conduct interviews with a representative sample of the primary care clinics in our randomized trial. Our sample included 3 small (1-3 health care providers) and 5 large (>3 health care providers) clinics. The sample included clinics that continued use of mPATH throughout the 6-month implementation period and those that discontinued use. Characteristics of the 8 clinics selected for interviews are shown in Table 1.

**Table 1:** Clinic characteristics.

Clinic	Size	High or low touch	Number of clinical personnel	Used mPATH for 12 months	Discontinued mPATH use before 12 months
A	Large	High	26	✓	
B	Small	High	8	✓	
C	Large	High	23	✓	
D	Large	High	14		✓
E	Small	Low	12	✓	
F	Large	Low	16		✓
G	Large	Low	16		✓
H	Small	Low	12		✓

### Interview Development

Semistructured interview guides were developed by the investigators and pretested to ensure clarity. Separate interview guides were developed for (1) front desk or administrative staff and (2) clinical personnel or practice managers. Because the front desk and administrative staff did not interact with the mPATH-CRC program, their interviews only asked about mPATH-CheckIn, whereas the clinical personnel and practice manager interviews included questions about both mPATH-CheckIn and mPATH-CRC. The interview guides were designed to collect descriptive data on the interviewee and explore the following constructs: (1) overall assessment of the effectiveness of mPATH-CheckIn for screening for depression, fall risk, and home safety and effectiveness of mPATH-CRC for CRC screening; (2) perceived barriers and facilitators as related to mPATH and clinic flow; (3) impact of mPATH on screening and clinic operations; (4) resources or training that were provided to the practice to support implementation; (5) program champion and leadership functioning; and (6) scale-up and dissemination. Because the trial took place during the COVID-19 pandemic, all interviewees were also asked how COVID-19 affected the implementation of mPATH.

### Data Collection, Management, and Analyses

Study personnel identified potential interviewees in collaboration with each practice’s clinic champion, a clinical staff member who served as the primary contact and was responsible for promoting mPATH internally and encouraging its adoption. Because we had previously conducted extensive usability testing of the app with patients, this study focused on health care providers and clinic staff [18]. To avoid influencing the randomized trial results, interviews were conducted after 1 year of implementation data collection was completed or after they stopped using the program, if earlier. All interviews were conducted via telephone by a single independent project staff member with no prior contact with participants. Interviews were audio-recorded, transcribed, deidentified, and reviewed. Transcripts were imported into ATLAS.ti (Scientific Software Development GmbH) to store and manage the data. Two study team members (AR and MC) inductively developed a codebook to identify meaningful categories in the dataset. Each transcript was independently coded by the 2 study team members, who met periodically to resolve discrepancies and revise the codebook as needed. Response saturation was identified when interview responses were repetitive rather than providing new information. The interview data were analyzed, and differences among participant groups (health care providers, nursing staff, clinic managers, and front desk staff) were labeled. Data within each category were abstracted and synthesized into themes. Themes were determined inductively by their prevalence and salience in the data per the principles of thematic analysis.

### Ethical Considerations

The Wake Forest University School of Medicine Institutional Review Board approved this study with a waiver of signed informed consent (IRB00048919). This study was also approved by the University of Kentucky Office of Research Integrity under a reliance agreement. Participants were provided with a study information sheet at the time of interview scheduling describing the purpose of the study and their rights to participate; risks and benefits and financial disclosures were declared. Participants provided verbal consent at the beginning of the interview to proceed and have their responses recorded. Interviews were conducted in private offices for confidentiality, and any participant or staff identifiers that were used in the interview discussion were removed from the transcripts. All interviewees were offered a US $50 gift card as compensation for their time.

## Results

### Overview

A total of 33 interviews were completed between September 2021 and April 2023 with health care providers (n=8, 24.2%), clinic managers (n=9, 27.3%), nursing staff (n=8, 24.2%), and front desk staff (n=8, 24.2%). Interviews averaged 25 minutes in length. Study staff identified the most prevalent facilitators of and barriers to mPATH implementation according to the methods described above. While some implementation facilitators and barriers were common to both mPATH-CheckIn and mPATH-CRC, there was some divergence of opinions on the 2, as described below.

### Implementation Facilitators

#### Staff and Health Care Provider Buy-In

Staff buy-in was identified as a significant factor in the success of mPATH implementation. Clinic staff were initially hesitant about implementing a new program and new workflows to accommodate mPATH, but this concern was alleviated with experience using the program:

I think that the biggest challenge would be, people worried about their workflow, and doctors are like everybody else. They do a lot of things just because that’s the way they do it. Implementing anything new maybe makes everyone a little nervous, but I think in general, everybody felt the way I did, that it didn’t disrupt workflows any significant way, so it was probably scary at the beginning, and didn’t prove to be much trouble, and once you do a new thing for a little while, you are okay.

Clinic A; health care provider

I think the number one most important factor was the—for lack of a better term—buy-in by the docs and the staff. Initially, folks were very enthusiastic about it.

Clinic D; health care provider

Buy-in from front desk staff was noted as important in mPATH-CheckIn because they were responsible for cleaning, storing, charging, and distributing mPATH-CheckIn tablets; instructing patients on use; answering patient questions; and preparing the tablets for each patient. Health care provider buy-in was also very important for the implementation of mPATH-CRC as they felt responsible for CRC screening in their eligible patients. Nursing staff bridged both mPATH-CheckIn and mPATH-CRC implementation workflows, and buy-in from them was important for smooth implementation and transition between both apps. mPATH-CheckIn had the most impact on nursing staff because it improved their workflow by conducting the required check-in screening, which, in turn, increased their buy-in to the program.

#### Training and Support

Interviewees found the in-clinic training sessions to be effective, especially the “hands-on” aspect of being able to test the tablets:

Yes, when we got trained on it, it was, basically, real-time training, and so she [mPATH research team staff member] would come in and help us on a day when we had patients. She walked through one patient with us, and just be there, step by step.

Clinic E; management

#### Leadership Support and Champion

Before implementation, each clinic identified a champion to lead implementation efforts. The practice manager or clinical coordinator was often identified as the champion. Staff recognized the importance of the clinic champion role and felt that mPATH implementation was well supported at the clinic leadership level.

#### Efficiency, Workflow, and Time Savings

Interviewees agreed that mPATH-CheckIn was a significant time saver and improved workflow during patient check-in and rooming, as patients answered questions directly into the tablet and answers were transferred from the tablet directly into the EHR:

Oh, ’cause it saves so much time between them already answering them, and they just pull it over into our system. Versus me going through each question myself and having to put those answers in. I just pull it over in there, and I didn’t have to go into any of those questions and open it up and ask.

Clinic G; nurse

Interviewees agreed that mPATH-CheckIn standardized and automated the assessment process and allowed more patients to be screened for depression, fall risk, and household violence than with previous workflows:

I do feel like it’s made the screening part much easier and faster, and we’ve been able to screen more patients...Typically, we wouldn’t get the chance to ask the questions just because of time and things like that. Typically, now they just do it in the waiting room when they’re checking in. I feel like a large majority of our patients are getting screened now.

Clinic E; health care provider

#### Patient Openness

Patient openness was noted by a significant number of interviewees as an important benefit of mPATH-CheckIn, specifically regarding depression questions. Staff perceived that asking depression questions on the tablet instead of verbally helped patients be more open and forthcoming with their answers. On the basis of interview responses, more patients with depression symptoms were identified through the tablet-based depression questionnaire than through previous procedures:

I think it actually helps the patient sometimes. It’s easier for them just to answer the question on the [tablet] rather than verbalize it ’cause sometimes they’re embarrassed to verbalize it to someone in-person. I think it’s easier for them just to answer it on the [tablet].

Clinic C; nurse

#### Patient Knowledge

Interviewees overall felt that the mPATH-CRC module with its educational video improved patient knowledge of CRC screening and the screening options available. It helped clarify the importance of screening, generate questions, and open the discussion when the health care provider entered the examination room:

I think that the content behind...[mPATH-CRC]...of giving our patients a visual, kind of audio/video on education with colon cancer screening, why they should be screened, and how they can do that is really helpful because I do that education with my patients, but some people learn better in a different manner, so that gives them a more visual or auditory way to learn. Then, they have, of course, the opportunity to ask more questions.

Clinic F; health care provider

#### Potential Impact on CRC Screening

Interviewees expressed that they believed that mPATH-CRC helped increase CRC screening or at least had the potential to increase screening:

I think the people that were on the fence of doing any type of screening, I think it did help do some screening. I think before our people said, nope, I’m not doing that—and I think after the mPATH, more people were open toward it.

Clinic C; health care provider

### Implementation Barriers

Analysis of the interviews also identified barriers to implementing mPATH in the clinics.

#### COVID-19 Pandemic–Related Barriers

Interviewees acknowledged that there were intervals of time when they did not use mPATH due to the pandemic or that there were protocols for disinfecting the mPATH tablets that slowed implementation:

...some of the patients, especially with COVID, would complain that they didn’t want to touch it (the tablet), so for a while, we could not use it at all, but now that we’ve added it back, some patients like it, and some patients absolutely refuse to do it.

Clinic A; management

However, not all clinics reported being affected by the pandemic:

I don’t think it affected it a whole lot. We sterilize our live stuff anyway. The only extra thing was more sterilization, but COVID really didn’t affect it a lot. We don’t really give the iPad to sick patients. None of those sick visits get the iPads, so it really didn’t affect it as much. Now, of course, patient volume was down during COVID, so we didn’t use it as often but other than that.

Clinic C; management

#### Technical Issues and EHR Limitations

Although, overall, interviews indicated that mPATH-CheckIn improved workflow, several interviewees (clinics B and G) voiced concerns about occasional data transmission errors between mPATH-CheckIn and the EHR:

There seemed to be an intermittent problem with the Medicare wellness visit when we used the mPATH for check-in. It would pull in some erroneous values. We weren’t able to figure out if that was part of mPATH or something else...

Clinic G; health care provider

Typically, when an issue arose where data were missing, staff would revert to their previous workflow of asking patients the mPATH-CheckIn questions verbally and then manually entering the data into the EHR.

There was also a perception that mPATH failed to recognize patients who received CRC screening outside the health system. Two clinics (A and F) with high numbers of patients who received screening outside the health system reported issues with mPATH-CRC properly identifying patients’ CRC screening status:

...almost all of our people are screened with outside of [University] system, and mPATH can’t see it. mPATH almost always tells me my patient hasn’t had a screen, and I just have to look anyway and see that they have, and then I have to make sure that it’s documented in a way that’s acceptable to our system, but being on the periphery like this, I don’t think mPATH stops me from basically having to do that same process and look. It always tells me they don’t have it, and most of them do. If our patients were getting it through a [University] gastroenterologist and it was in our [EHR], I think that would be different, but it doesn’t seem to identify the ones of ours that are done in community clinics, outside of our network.

Clinic A; health care provider

This added to staff burden as the health care provider or nurse had to double-check or verify the screening status of each patient before giving the patient the mPATH-CRC tablet.

#### Lack of Need or Demand for the Program

Specific to mPATH-CRC, while interviewees were generally positive about the potential of the program to improve patient knowledge and potentially help increase screening rates, most reported that they infrequently used mPATH-CRC, primarily due to patient eligibility factors. Clinics reported that many of their patients were outside of screening age or were up-to-date with CRC screening due to a colonoscopy within the previous 10 years.

#### Efficiency, Workflow, and Time Barriers

There were some barriers noted that impacted workflow, specifically the transition between the mPATH-CheckIn and mPATH-CRC apps. As patients were given mPATH-CheckIn in the waiting room and then mPATH-CRC after they were taken back to an examination room, any delays at the front end of the workflow had the potential to carry over to the back end. This particularly occurred with patients with limited technical skills who required more time with the tablets:

I feel like, for our patients that were more technologically inclined, it, in some cases, was able to help facilitate that data gathering more quickly. For other patients that were less technologically inclined, we found that often they hadn’t made it through or had only partially made it through the questionnaire. Then the MAs [medical assistants] would go back in and redo the process.

Clinic G; health care provider

For mPATH-CRC specifically, several clinics said that the 5-minute screening decision aid video was too long and had a negative impact on workflow, especially health care provider workflow:

Because I personally liked that they can watch the video, but some of our providers do not like it, because it obviously delays them going into the room a little bit. That’s the main hiccup that we’ve had is just the providers have to wait however many minutes. I don’t remember how many minutes the video is, but they haven’t wanted to do that.

Clinic A; nurse

### Overall Recommendation for mPATH

Health care providers and staff from 4 clinics reported that they were still using mPATH at the time of their interviews, whereas 1 clinic reported continuing use of mPATH-CheckIn only. Interviewees from 3 clinics reported that they had discontinued use of the program at the time of their interviews. However, those who had discontinued the program reported that they liked it and would have continued using it if it were up to them individually. The decision to discontinue the program was often made by other clinic leadership who were not interviewed or was a group decision among health care providers and staff as a whole. All interviewees were positive about recommending that other clinics at least try mPATH to determine whether it was a good fit for them.

## Discussion

### Principal Findings

We found that the opinions of the clinical staff and health care providers about implementing the tablet-based screening program were impacted by staff buy-in, how well the program was incorporated into existing workflows, and the additional value it delivered. Concern related to technical issues was the main barrier described by interviewees. Those interviewed considered mPATH-CheckIn to be beneficial as a time saver and as a means to streamline and improve efficiency in the patient data collection process at check-in by providing an easy-to-use technology for increasing their quality metrics for depression, fall risk, and household violence and abuse. These factors seemed to serve as motivators for clinics to use mPATH, especially the clinical staff.

While the clinic staff interviewed expressed appreciation for the concept of mPATH-CRC “in theory,” it was challenging to implement due to a variety of factors. Health care providers were most opinionated about mPATH-CRC compared to nursing staff, perhaps due to health care providers expressing their feelings of “ownership” and “responsibility” for ensuring that their patients were up-to-date with CRC screening. The nursing staff interviewed had a very limited or peripheral role in the CRC screening process—primarily reviewing patient screening history and notifying health care providers if the patient was due for screening. The health care providers interviewed seemed more resistant to changing their current workflows to accommodate mPATH-CRC and often indicated that “they do what mPATH-CRC does anyway,” seeing it as a duplication or add-on to their existing preferred process. Another major factor in implementing mPATH-CRC was the belief that most patients were already up-to-date with CRC screening and did not need mPATH-CRC. Finally, mPATH-CheckIn was intended to be used with every patient aged >18 years, and mPATH-CRC was intended to be used just with those patients aged >50 years who were due for screening, reflecting screening guidelines in place at the start of the study. The need to differentiate patients by age may have impacted clinics’ comfort level with the mPATH-CRC program as it was not routinely used for all adult patients.

Our findings are consistent with those of other trials that have reported difficulty implementing digital health interventions in primary care environments. In a hybrid implementation-effectiveness study of a digital health program to support patients with substance use disorders in federally qualified health centers, the program was offered to fewer than 10% of eligible patients [22]. Mei and Vaez [23] implemented electronic screening for substance use and mental health in a community-based university health system–affiliated primary care practice; although screening completion increased, the absolute improvement was modest at 9%. Similarly, Yakovchenko et al [24] implemented an automatic SMS text messaging program to help with hepatitis C virus medication adherence in Veterans Health Administration primary care clinics, with fewer than 20% of approached patients progressing to active use of the system.

Despite being conducted in markedly different health system contexts (federally qualified health centers, the US Department of Veterans Affairs health system, and a university-affiliated primary care practice), all 3 studies reported similar barriers to implementation, including workflow disruptions, technical challenges, and increased clinician burden. Each study also identified perceived time savings, leadership support, and staff engagement as important facilitators of adoption. Consistent with these findings, published literature reviews have repeatedly identified workflow integration as one of the strongest determinants of digital health implementation, with tools perceived to save time more likely to be adopted and those perceived to add burden less likely to be used [12,25-27].

Our study extends this literature by highlighting the importance of routinization. Participants specifically mentioned that the mPATH-CRC program was less likely to be used because it was only administered to age-eligible patients who needed screening. In contrast, the mPATH-CheckIn program was seen as easier to administer because it was designed to be used with every patient at every visit. In addition, because our study was conducted during the COVID-19 pandemic, our findings yielded unique insights into how public health concerns can affect implementation. Approximately half of the interviewees reported that patients were concerned about germs and touching the tablets. Although clinics used antibacterial tablet cases and implemented cleaning protocols that were communicated to patients, these reassurances were not always adequate to allay concerns. In addition, pandemic-related workflows, such as restricted tablet distribution to patients who were well, limited implementation in some clinics.

### Limitations

The program was implemented in clinics all within the same health system and the same EHR system, which may have impacted opinions, perceptions, and technical issues regarding connectivity and integration. While we sought candid interview responses by ensuring confidentiality and using an external interviewer, social desirability bias may have affected responses. The fact that the study was conducted in the same health system to which the research team is affiliated may have also impacted responses. As stated previously, we conducted interviews after all quantitative implementation outcome data were collected, which was 1 year after program launch. This time delay may have impacted respondents’ memories and perceptions of the initial training and implementation activities. Finally, because we previously found that the program had excellent patient usability [13,15], and as staff were responsible for administering the program, we did not include patients in our interviews. Patient-level factors not identified by clinical staff may have affected implementation.

### Conclusions

When implementing tablet-based digital health programs in primary care clinics, demonstrating the programs’ ability to improve care, fit into usual workflows, and ideally save time is critical. Providing hands-on training before or at the start of implementation and identifying a suitable clinic champion can help increase buy-in and comfort with the program. For decision aids related to cancer or other health screenings, the demand for the program should be weighed against potential barriers to ensure that the program can be implemented and sustained effectively. Review of potential implementation facilitators and barriers can help maximize the uptake of digital health programs and enhance continued use.

## Abbreviations

CRC: colorectal cancer
EHR: electronic health record

## Data Availability

Data sharing is not applicable to this paper as no datasets other than transcripts were generated or analyzed during this study.
